# Skin Conductance as an Index of Alexithymic Traits in the General Population

**DOI:** 10.1177/00332941211005118

**Published:** 2021-03-31

**Authors:** Lydia J. Hickman, Connor T. Keating, Ambra Ferrari, Jennifer L. Cook

**Affiliations:** School of Psychology, 1724 University of Birmingham, UK; Donders Institute for Brain, Cognition and Behaviour, Radboud University, the Netherlands; School of Psychology, 1724 University of Birmingham, UK

**Keywords:** Alexithymia, physiological arousal, skin conductance, subjective arousal, objective arousal

## Abstract

Alexithymia concerns a difficulty identifying and communicating one’s own emotions, and a tendency towards externally-oriented thinking. Recent work argues that such alexithymic traits are due to altered arousal response and poor subjective awareness of “objective” arousal responses. Although there are individual differences within the general population in identifying and describing emotions, extant research has focused on highly alexithymic individuals. Here we investigated whether mean arousal and concordance between subjective and objective arousal underpin individual differences in alexithymic traits in a general population sample. Participants rated subjective arousal responses to 60 images from the International Affective Picture System whilst their skin conductance was recorded. The Autism Quotient was employed to control for autistic traits in the general population. Analysis using linear models demonstrated that mean arousal significantly predicted Toronto Alexithymia Scale scores above and beyond autistic traits, but concordance scores did not. This indicates that, whilst objective arousal is a useful predictor in populations that are both above and below the cut-off values for alexithymia, concordance scores between objective and subjective arousal do not predict variation in alexithymic traits in the general population.

## Introduction

Alexithymia is defined as a difficulty in identifying and describing one’s own emotions, and a tendency towards externally-oriented thinking (Bagby et al., 1994; Nemiah et al., 1976). It is typically measured by self-report scales; a commonly used measure is the Toronto Alexithymia Scale (TAS; Bagby et al., 1994), a questionnaire consisting of 20 statements such as “I am often confused about what emotion I am feeling” and “I find it hard to describe how I feel about people”. With relationships emerging between alexithymia and socio-cognitive processes (e.g., emotion recognition and production; empathy; Bird et al., 2010; Brewer et al., 2015; Cook et al., 2013; Trevisan et al., 2016), and mental health more broadly (Norman & Borrill, 2015; Ogrodniczuk et al., 2011), alexithymia is of increasing importance for higher cognition and health respectively. A key issue, however, concerns the underlying psychophysiological mechanisms that give rise to alexithymic difficulties in identifying and describing emotions. Elucidating the underlying psychophysiological mechanisms is not only important for gaining insight into the pathways that contribute to such challenges, but also may result in the development of objective measures of alexithymia which draw upon physiological markers of arousal. Indeed, a key problem for this field is that the measurement of alexithymia relies almost exclusively on self-report questionnaires that require participants to reflect on the difficulties they have in reflecting on their own emotions (Vorst & Bermond, 2001). Consequently, an objective measure of alexithymia is much sought after.

Extant studies of the psychophysiological mechanisms underlying alexithymic traits have drawn an important distinction between objective and subjective arousal (e.g., Gaigg et al., 2018). The former concerns a bodily reaction to a contextual cue, whereas the latter concerns a subjective judgement about one’s own arousal level. Whereas subjective arousal is assessed by asking participants to reflect on their physiological state, objective arousal can be assessed using a variety of methods including heart rate recordings (Eastabrook et al., 2013; Papciak et al., 1985; Pollatos et al., 2011; Stone & Nielson, 2001), blood pressure (Papciak et al., 1985) and electromyography (Papciak et al., 1985). The most common method for assessing objective arousal is to record skin conductance in response to arousing stimuli. Skin becomes a better conductor of electricity when an individual is physiologically aroused thus it is typically observed that when participants are exposed to high, compared to low, arousal stimuli their skin conductance response reaches a higher maximum peak (e.g., Gaigg et al., 2018), and/or maintains a higher average level of activity throughout the stimulus presentation interval (e.g., Pollatos et al., 2011). By drawing this distinction between objective and subjective arousal, the literature has made progress in understanding whether the difficulties experienced by individuals *with clinically significant levels of alexithymia* are due to broader impairments in mental state reasoning (Moriguchi et al., 2006) and/or atypical physiological arousal.

Studies of objective and subjective arousal have highlighted at least two mechanisms thought to contribute to emotion identification and communication problems in populations with clinically significant levels of alexithymia: 1) altered levels of objective emotional arousal, and 2) reduced awareness of otherwise preserved emotional arousal (see Vorst & Bermond, 2001 for further discussion). A small but burgeoning literature has found evidence consistent with both the former mechanism (note that there is evidence to suggest both hyper-arousal (Eastabrook et al., 2013; Papciak et al., 1985; Stone & Nielson, 2001) and hypo-arousal (e.g., Gaigg et al., 2018; Pollatos et al., 2011; Roedema & Simons, 1999) and the latter mechanism (Eastabrook et al., 2013; Gaigg et al., 2018; Papciak et al., 1985; Pollatos et al., 2011; Stone & Nielson, 2001). A study by Gaigg et al. (2018) is particularly notable because the design enabled the authors to calculate individual participant scores corresponding to the two aforementioned mechanisms: objective arousal responses and the concordance between subjective and objective arousal. Thus, this facilitated investigation of whether self-reported difficulties with emotion identification and description are predicted by either mechanism or a combination of the two mechanisms. Specifically, Gaigg et al. asked participants to rate their subjective arousal responses to images from the International Affective Picture System (IAPS; a set of colour photographs with normative emotion ratings; Lang et al., 2008). Concurrently, objective arousal responses to each image were measured in terms of skin conductance responses. Gaigg et al. observed that self-reported alexithymic traits were predicted by altered levels of objective arousal *and* independently with a reduced correlation between subjective and objective arousal.

It is important to note that the majority of studies investigating these two mechanisms in alexithymia have adopted the approach of assessing group differences between alexithymic and non-alexithymic individuals (e.g., Eastabrook et al., 2013; Papciak et al., 1985; Pollatos et al., 2011; Roedema & Simons, 1999; Stone & Nielson, 2001). This relies on a categorical view of alexithymia and assumes a cut-off point for alexithymic traits. Conversely, the method adopted by Gaigg et al. (2018) of creating individual objective arousal and concordance scores allows for a continuous analysis approach. Here, relationships between levels of alexithymic traits and the extent of psychophysiological differences can be observed. This is of importance as such individual differences in identifying one’s own emotions have been found to predict important functions such as sleep quality (Murphy et al., 2018) and mental health (Norman & Borrill, 2015; Ogrodniczuk et al., 2011).

To date it is unclear whether the mechanisms that underpin emotion identification and communication problems in alexithymia, also underpin variation in the general population. In the sample studied by Gaigg et al. (2018), only 42% could be categorised as non-alexithymic (according to Deborde et al. (2008) suggested cut-offs). Furthermore, 50% of Gaigg and colleagues’ sample also had a co-morbid autism diagnosis. Co-occurring autism was a relevant feature of Gaigg et al’s design. Building on literatures that document atypically high rates of alexthymia in autistic populations^1^ (50% of autistic individuals are alexithymic compared to 5% of the general population (Kinnaird et al., 2019)), and which demonstrate that alexithymia can account for atypicalities in emotional processing and empathy in autistic individuals (Bird et al., 2010; 2011; Cook et al., 2013), Gaigg et al. aimed to test the prediction that the co-occurrence of alexithymia in an autistic sample is associated with an impairment in the subjective awareness of otherwise intact physiological arousal responses (as reported by Ben Shalom et al., 2006). Gaigg et al.’s results, however, contradicted their hypothesis: in their sample, alexithymia was associated with atypical objective arousal *and* impaired subjective awareness. Indeed, these results align with a body of literature documenting atypical objective arousal responses in autism (e.g., Dijkhuis et al., 2019; Hirstein et al., 2001; Hubert et al., 2009; Mathersul et al., 2013). Consequently, whilst Gaigg et al. addressed an important question that has advanced our understanding of the interplay between autism and alexithymia, the sample they recruited is potentially biased towards individuals who are more likely than other members of the general population to exhibit atypicalities in arousal responses. Thus, to gain an unbiased understanding of whether the mechanisms that underpin emotion identification and communication problems in alexithymia also underpin variation in the general population, it is important to recruit a non-clinical general population sample. Furthermore, since there is evidence that autistic symptomatology is correlated with both alexithymic traits (Hobson et al., 2020) and atypical physiological arousal (e.g., Dijkhuis et al., 2019; Hirstein et al., 2001; Hubert et al., 2009; Mathersul et al., 2013), there is a risk that, if autistic traits are not controlled for, relationships observed between objective arousal and alexithymic traits are mediated by autistic traits. Consequently, to understand these relationships in the general population, it is not only important to recruit a non-clinical general population sample, but autistic traits must also be controlled for.

The current study investigated whether individual differences in emotion identification and description *in the general population* are underpinned by variation in 1) levels of objective emotional arousal and/or 2) subjective awareness of objective arousal (“concordance scores”). To do so we employed the procedure developed by Gaigg et al. which enabled us to assess individual differences in subjective and objective arousal. Participants, who comprised a random sample of the general population, completed an arousal estimation task based on Gaigg et al. (2018). Self-reported difficulties identifying one’s own emotions were indexed with the Toronto Alexithymia Scale (TAS; Bagby et al., 1994). Participants completed the Autism Quotient (AQ; Baron-Cohen et al., 2006) to control for levels of autistic traits. Objective arousal was calculated as mean skin conductance across all trials and concordance scores as the correlation between objective arousal (skin conductance) and subjective arousal (self-reported arousal). Linear models were employed to assess the extent to which alexithymic traits could be predicted by a) objective arousal and b) concordance scores, whilst controlling for autistic traits. We predicted that, as one would expect for a highly alexithymic sample, emotion identification difficulties would be associated with altered objective arousal *and* a reduced correlation between subjective and objective arousal.

## Method

### Participants

An a priori power analysis calculated with G*power (Erdfelder et al., 1996) using data from Gaigg et al. (2018) (effect size = 0.46, alpha level = .05) determined that a minimum of 32 participants were required to achieve a power level of 0.80. This power level, convention in the field of psychology, was based on recommendations that the probability of Type 2 errors – beta – should not exceed four times the probability of Type 1 errors – alpha (Cohen & Wolman, 1965). Thus, given the conventional alpha level of 0.05 and a consequent recommended beta level of 0.2, a 0.8 power level was used (power = 1 – beta). We recruited 43 healthy participants via the University of Birmingham Research Participation Scheme. A total of 35 participants (27 female, 7 male, 1 undisclosed) were included in the analysis due to 8 participants not providing complete data for the arousal estimation task and questionnaires. The sample had a mean age of 21 years (standard deviation [SD] = 2.57). All participants gave fully informed consent and received either course credits or money (£8 per hour). The experimental procedure was approved by the local Research Ethics Committee (ERN 16-0281AP5).

### Arousal estimation task

#### Stimuli

A total of 60 images from the International Affective Picture System (IAPS; Lang et al., 2008) were selected for use in the arousal estimation task, each with pre-defined arousal and valence ratings generated from ratings by 100 individuals made on 9-point rating scales (Lang et al., 2008). The images covered a wide range of valence (mean[SD] = 4.93[2.23]) and arousal ratings (mean[SD] = 4.88[1.81]), aiming to elicit variation in participants’ reactions during the task. In order to achieve systematic variation in the images used, 20 images defined as positive (10 high arousal and 10 moderate arousal), 20 as negative (10 high arousal and 10 moderate arousal) and 20 as neutral (all low arousal) were selected. An additional 6 images representative of the images used in the experimental trials were selected for use in the practice trials. See Appendices 1 and 2 for IAPS numbers, arousal ratings and valence ratings for the images used in the practice and experimental trials respectively.

#### Procedure

**Figure 1.**
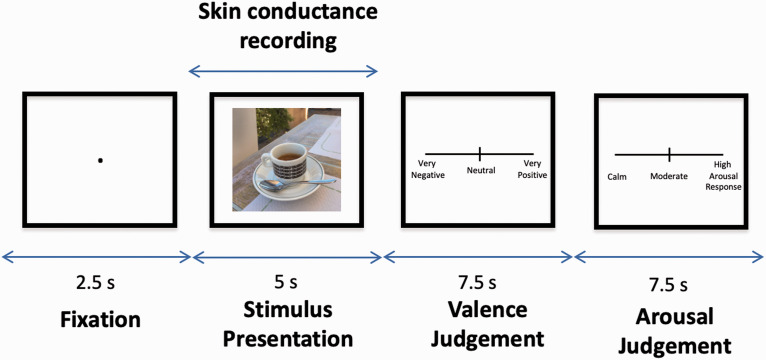
The structure of each trial within the arousal estimation task. Each trial involved a fixation, stimulus presentation, valence judgement and arousal judgement. The image used in the diagram is a placeholder and not an IAPS image presented to participants in the experiment. ‘S’ denotes time in seconds of each period of the trial.

Following a practice of 6 trials, participants viewed 20 positive, 20 negative and 20 neutral IAPS images. During each trial, the stimulus was presented for 5 seconds with a preceding fixation dot lasting for a duration of 2.5 seconds. Skin conductance was recorded concurrently during the 5 second stimulus presentation window using a Biopac MP36R, with disposable isotonic gel electrodes attached to the distal phalanges of participants’ index and middle fingers on their non-dominant hand. After viewing each image, participants were asked to rate how positive or negative it was on a sliding scale ranging from ‘very negative’, to ‘neutral’, to ‘very positive’ (valence). They were then asked to rate their arousal level in response to the image on a sliding scale ranging from ‘calm’ to ‘moderate’ to ‘high arousal response’ (arousal). For each question, participants had 7.5 seconds to make a response. The scale remained on the screen for the full 7.5 seconds irrespective of the response time. The structure of each trial is displayed in Figure 1.

#### Data processing

Responses to the valence and arousal questions were converted to ratings out of 100 for each trial. Skin conductance (SC) data were analysed using Acqknowledge Software. Various skin conductance indices have been used in the literature to index arousal, generally focusing on the peak or magnitude of the responses during stimulus presentation (e.g., Gaigg et al., 2018), or the average levels of activity during the interval in which a stimulus is presented (e.g., Pollatos et al., 2011). In the current study, we created indices of both peak (‘max’) and average (‘mean’) skin conductance levels in response to stimulus presentation. Following the smoothing of the data using a 2 Hz low pass filter to remove noise, two SC values were calculated for each trial: the mean SC level within the 5 second stimulus presentation window (SC-mean) and maximum SC value observed within the 5 second stimulus presentation window (SC-max). The former draws upon recent suggestions that average skin conductance levels may be useful in distinguishing responses to stimuli (e.g., Sugimine et al., 2020), and the latter reflects the peak value recorded during stimulus presentation.

### Questionnaires

Participants completed the 20-item TAS to index alexithymic traits, with individuals categorised as non-alexithymic if their score fell below 51 (Bagby et al., 1994). The TAS has good internal consistency and test-retest reliability (α ≥ 0.7; r ≥ 0.7; Bagby et al., 1994; Taylor et al., 2003) and is the most commonly used measure of alexithymic traits. The 50-item AQ (Baron-Cohen et al., 2006), a questionnaire with strong psychometric properties including internal consistency and test-retest reliability (α ≥ 0.7; r ≥ 0.8; Stevenson & Hart, 2017), was employed to control for autistic traits in the general population. The order of the arousal estimation task and questionnaires was counterbalanced.

### Score calculations

Following the work of Gaigg et al., our primary measures were *average SC* – as an index of objective arousal – and *concordance score* – as an index of the correlation between subjective and objective arousal. Average SC (microsiemens) was calculated as the mean of the SC data across all trials. Concordance scores were calculated as the Spearman’s correlation coefficient between participants’ self-reported arousal rating on each trial and SC during the 5 second stimulus presentation windows. The two scores were calculated using both SC-mean and SC-max data.

Considering evidence that stimulus valence can modulate arousal responses (Bradley & Lang, 2000), concordance between arousal ratings and objective arousal responses may be affected by the valence of the stimulus. Thus, we sought to create an index of concordance which controlled for the impact of the valence of the stimuli in the relationship between subjective and objective arousal. As a result, *partial concordance scores* were calculated as the Spearman’s partial correlation coefficient between participants’ self-reported arousal ratings and objectively measured SC on each trial controlling for participants’ self-reported valence ratings on each trial. Again, separate indices were calculated using the SC-mean and SC-max data.

TAS scores were calculated as a sum of participants’ responses, using reverse scoring where appropriate, with a maximum possible score of 100 reflecting the highest level of alexithymic traits. The AQ was scored as the sum of participants’ responses, using reverse scoring where appropriate, with a maximum score of 50 reflecting the highest level of autistic traits.

### Analyses

To test our hypothesis that alexithymic traits would be associated with altered objective arousal *and* a reduced correlation between subjective and objective arousal, we employed two linear models with AQ score, average SC and concordance score as predictors and TAS score as the dependent variable. All variables were z-scored. Model 1 used SC-mean data, whilst Model 2 used SC-max data. Subsequently, linear models were implemented which instead used the partial concordance score (which controls for the effect of stimulus valence) in place of the standard concordance score. Again, one model used SC-mean data (model 3) and another used SC-max data (model 4). Final models were then conducted, predicting TAS score with the significant variables identified in models 1–4, with separate models for SC-mean and SC-max data.

## Results

Descriptive statistics for the 35 participants who completed all tasks were as follows: TAS score (mean[SD] = 42.63[10.20]; 77% of the sample were categorised as non-alexithymic), AQ score (mean[SD] = 14.74[7.04]), average SC-mean (mean[SD] = 5.03[0.30]), average SC-max (mean[SD] = 5.20[0.41]), SC-mean concordance score (mean[SD] = 0.05[0.16]), SC-max concordance score (mean[SD] = 0.06[0.17]), SC-mean partial concordance score (mean[SD] = 0.04[0.14]), SC-max partial concordance score (mean[SD] = 0.05[0.16]).

To investigate whether variation in emotion identification and description was predicted by 1) objective arousal and/or, 2) subjective awareness of objective arousal, linear models predicting TAS score with average SC and concordance score were employed, using either SC-mean data (model 1) or SC-max data (model 2). AQ score was included in the model to control for variation associated with autistic traits. For both models, AQ score and average SC were significant positive predictors of TAS score, whereas concordance score was not a significant predictor (Table 1). Thus, increased alexithymic traits can be predicted by increased autistic traits and increased objective arousal. Model 1 had an adjusted R^2^ value of 0.39, meaning that 39% of the variance in TAS scores was accounted for. The addition of average SC-mean – our index of objective arousal – in model 1 resulted in an R^2^ change of 0.19 (significant model improvement: *F*(1, 31) = 10.53, *p* = .003) meaning that an additional 19% of the variance in TAS was accounted for by including SC-mean in the model. Model 2 had an adjusted R^2^ value of 0.32, meaning that 32% of the variance in TAS scores was accounted for, and an R^2^ change of 0.13 (13% variance accounted for) resulting from the addition of average SC-max (significant model improvement: *F*(1, 31) = 6.61, *p* = .015). Models 1 and 2 therefore demonstrate that, in a general population sample wherein autistic traits are controlled for, objective arousal is a significant positive predictor of variation in emotion identification and description as measured by the TAS.

**Table 1.** The results of six linear models in which TAS score was predicted by AQ score, average SC, and concordance score (models 1 and 2), AQ score, average SC, and partial concordance score (models 3 and 4), or AQ score and average SC (models 5 and 6).

Model	Variable	Estimate	Standard error	t value	*p* value
1	AQ score	0.62	0.14	4.40	<.001
	Average SC-mean	0.45	0.14	3.25	.003
	SC-mean concordance score	0.19	0.14	1.41	.170
2	AQ score	0.60	0.15	4.06	<.001
	Average SC-max	0.38	0.15	2.57	.015
	SC-max concordance score	0.15	0.14	1.05	.300
3	AQ score	0.62	0.14	4.42	<.001
	Average SC-mean	0.45	0.14	3.29	.003
	SC-mean partial concordance score	0.21	0.13	1.53	.136
4	AQ score	0.60	0.15	4.08	<.001
	Average SC-max	0.38	0.15	2.61	.014
	SC-max partial concordance score	0.16	0.14	1.13	.269
5	AQ score	0.59	0.14	4.16	<.001
	Average SC-mean	0.44	0.14	3.15	.004
6	AQ score	0.58	0.15	3.94	<.001
	Average SC-max	0.39	0.15	2.63	.013

SC-mean data were used for models 1, 3 and 5, and SC-max data were used for models 2, 4 and 6.

To probe whether a relationship between alexithymic traits and subjective awareness of objective arousal emerges when controlling for effects on arousal of the valence of the stimuli, two linear models were employed whereby partial concordance score was used in place of the standard concordance score. The two models used SC-mean data (model 3) and SC-max data (model 4) respectively. Again, AQ and average SC were significant positive predictors of TAS score in both models, and partial concordance score did not significantly predict TAS score (Table 1). Model 3 had an adjusted R^2^ value of 0.39; thus, the model accounted for 39% of the variance in TAS scores. The addition of average SC-mean in the model resulted in an R^2^ change of 0.19 (19% variance accounted for; significant model improvement: *F*(1, 31) = 10.80, *p* = .003). Model 4 had an adjusted R^2^ value of 0.33, meaning that 33% of the variance in TAS scores was accounted for, and an R^2^ change of 0.13 (13% variance accounted for) resulting from the addition of average SC-max (significant model improvement: *F*(1, 31) = 6.80, *p* = .014). These models demonstrate that, even after controlling for the valence of the stimuli, concordance between subjective and objective arousal was not associated with alexithymic traits; objective arousal remained a significant predictor.

Two final linear models were conducted wherein predictors that were non-significant in models 1–4 were dropped; this resulted in two models predicting TAS from AQ score and SC-mean (model 5), and AQ score and SC-max (model 6), respectively. These models enabled us to quantify the amount of variance in TAS explained by objective arousal when AQ is controlled for. All variables were significant positive predictors (Table 1). Models 5 and 6 accounted for 37% and 32% of the variance in TAS scores respectively (model 5: R^2^ = 0.37; model 6: R^2^ = 0.32). The addition of average SC-mean in model 5 explained 18% of variance in TAS scores (R^2^ change = 0.18; *F*(1, 32) = 9.90, *p* = .004), and the addition of average SC-max in model 6 explained 14% of variance (R^2^ change = 0.14; *F*(1, 32) = 6.92, *p* = .013). Prior to adding either average SC-mean or average SC-max, AQ accounted for 20% of variance in TAS scores (R^2^ = 0.20). These analyses demonstrate that AQ scores and average SC are significant positive predictors of TAS scores when concordance scores are not included in the model, and that average SC explains variance above and beyond autistic traits.

## Discussion

This study investigated whether alexithymic traits in the general population (as indexed by the TAS) are associated with altered objective arousal *and* a reduced correlation between subjective and objective arousal. Results from six linear models demonstrated that objective arousal (as indexed by average SC) was a significant positive predictor of TAS score. That is, individuals with a higher TAS score had greater levels of physiological arousal in response to the IAPS stimuli. This result is consistent with findings associating alexithymia with hyper-arousal (Eastabrook et al., 2013; Papciak et al., 1985; Stone & Nielson, 2001) and with predictions from the stress-alexithymia hypothesis (Martin & Pihl, 1985), which proposes that individuals with alexithymia “lack the affective awareness which would permit identification of a particular situation as stressful” and consequently experience stressful events more frequently and for longer periods of time. Here, we demonstrate that this particular mechanism (objective arousal) is likely to contribute to alexithymic traits in the general population, something that previous studies taking a group differences approach or recruiting highly alexithymic samples have not been able to show. In addition, average SC significantly predicted TAS scores regardless of whether SC-mean or SC-max was used, accounting for an additional 19% and 13%^2^ of the variance in TAS scores respectively; this demonstrates the utility of both indices in predicting alexithymic traits. These results pave the way for the development of objective measures of alexithymia which draw upon physiological markers of arousal in place of self-report. However, given that our best estimate is that 19% of the variance in alexithymic traits can be accounted for by skin conductance, future work may seek to combine multiple objective measures in order to more accurately predict alexithymic traits.

It should be noted that objective arousal significantly improved the prediction of TAS scores above and beyond autistic traits as measured by the AQ. This was demonstrated through the inclusion of AQ scores within the six linear models predicting TAS scores. More specifically, AQ accounted for 20% of the variance in alexithymic traits in our population and adding objective arousal indices to the model enabled us to account for an additional 18% and 14% of variance for SC-mean and SC-max respectively (see models 5 and 6). The importance of this aspect of the analyses is highlighted by previous studies which have shown correlations between alexithymic traits and autistic symptomatology (Hobson et al., 2020), and atypical objective arousal in autism (e.g., Dijkhuis et al., 2019; Hirstein et al., 2001; Hubert et al., 2009; Mathersul et al., 2013). Thus, there is a risk that correlations observed between objective arousal and TAS are mediated by autistic traits. Including AQ in our linear model enabled us to observe a significant relationship between average SC and TAS scores after removing variance associated with autistic traits. These results further strengthen the conclusion that average SC is a significant predictor of TAS scores in the general population.

In contrast to our prediction, TAS scores were not predicted by concordance scores between objective and subjective arousal, nor partial concordance scores between objective and subjective arousal which controlled for valence. Though reduced subjective awareness of objective arousal is a putative mechanism underpinning emotion identification and communication issues in the alexithymic population (Gaigg et al., 2018; Vorst & Bermond, 2001), we failed to find any evidence that this mechanism underpins variation in such traits in the general population. Thus, concordance scores do not appear to be a useful predictor of alexithymic traits in the general population. This result raises the possibility that concordance scores should not be viewed as a continuous marker but rather as a binary index separating alexithymic and non-alexithymic populations. Indeed, previous studies employing a group differences approach to concordance have presented evidence for intact concordance in the non-alexithymic group (Eastabrook et al., 2013; Papciak et al., 1985; Pollatos et al., 2011; Stone & Nielson, 2001). This leads us to consider that, whilst variation in alexithymic traits in the general population is correlated with objective arousal responses, the inability to reflect upon one’s own emotions may be viewed as a binary metric which highlights clinically significant levels of alexithymia.

Taken together, our findings raise doubts as to whether all psychophysiological mechanisms underpinning emotion identification and communication problems in alexithymic individuals also underpin such issues in the general population. Whilst objective arousal appears to be a useful predictor in populations that are both above and below the cut-off values for alexithymia, concordance scores between objective and subjective arousal do not predict variation in emotion identification and communication in the general population. Such findings are important given the growing emphasis on individual differences in alexithymic traits and their associations with mental health (e.g., Murphy et al., 2018; Norman & Borrill, 2015; Ogrodniczuk et al., 2011).

## Appendix 1: IAPS images used in the practice trials of the arousal estimation task

**Table:** 

Valence group	Arousal group	IAPS number	Valence mean	Valence standard deviation	Arousal mean	Arousal standard deviation
Negative	High	3130	1.58	1.24	6.97	2.07
Negative	Moderate	9181	2.26	1.85	5.39	2.41
Neutral	Low	7010	4.94	1.07	1.76	1.48
Neutral	Low	7161	4.98	1.02	2.98	1.99
Positive	High	4659	6.87	1.99	6.93	2.07
Positive	Moderate	2398	7.48	1.32	4.74	2.11

## Appendix 2: IAPS images used in the experimental trials of the arousal estimation task

**Table:** 

Valence group	Arousal group	IAPS number	Valence mean	Valence standard deviation	Arousal mean	Arousal standard deviation
Negative	High	9635.1	1.9	1.31	6.54	2.27
		3102	1.4	1.14	6.58	2.69
		9413	1.76	1.08	6.81	2.09
		3400	2.35	1.9	6.91	2.22
		6260	2.44	1.54	6.93	1.93
		6550	2.73	2.38	7.09	1.98
		3170	1.46	1.01	7.21	1.99
		3080	1.48	0.95	7.22	1.97
		3010	1.79	1.28	7.26	1.86
		3000	1.59	1.35	7.34	2.27
	Moderate	2205	1.95	1.58	4.53	2.23
		2301	2.78	1.38	4.57	1.96
		9561	2.68	1.92	4.79	2.29
		9830	2.54	1.75	4.86	2.63
		2141	2.44	1.64	5	2.03
		2799	2.42	1.41	5.02	1.99
		2053	2.47	1.87	5.25	2.46
		2710	2.52	1.69	5.46	2.29
		3185	2.81	1.52	5.48	2.18
		9043	2.52	1.42	5.5	2.41
Neutral	Low	7025	4.63	1.17	2.71	2.2
		7150	4.72	1	2.61	1.76
		7217	4.82	0.99	2.43	1.64
		2393	4.87	1.06	2.93	1.88
		7175	4.87	1	1.72	1.26
		2840	4.91	1.52	2.43	1.82
		7059	4.93	0.81	2.73	1.88
		7235	4.96	1.18	2.83	2
		7041	4.99	1.12	2.6	1.78
		7004	5.04	0.6	2	1.66
		7179	5.06	1.05	2.88	1.97
		2038	5.09	1.35	2.94	1.93
		7233	5.09	1.46	2.77	1.92
		7090	5.19	1.46	2.61	2.03
		5740	5.21	1.38	2.59	1.99
		2850	5.22	1.39	3	1.94
		7100	5.24	1.2	2.89	1.7
		7026	5.38	1.26	2.63	1.93
		5731	5.39	1.58	2.74	1.95
		7140	5.5	1.42	2.92	2.38
Positive	High	4660	7.4	1.36	6.58	1.88
		8180	7.12	1.88	6.59	2.12
		4698	6.5	1.67	6.72	1.72
		8370	7.77	1.29	6.73	2.24
		8186	7.01	1.57	6.84	2.01
		4668	6.67	1.69	7.13	1.62
		4220	8.02	1.93	7.17	2.69
		8185	7.57	1.52	7.27	2.08
		8492	7.21	2.26	7.31	1.64
		8030	7.33	1.76	7.35	2.02
	Moderate	2091	7.68	1.43	4.51	2.28
		2070	8.17	1.46	4.51	2.74
		1440	8.19	1.53	4.61	2.54
		2550	7.77	1.43	4.68	2.43
		2340	8.03	1.26	4.9	2.2
		7330	7.69	1.84	5.14	2.58
		8540	7.48	1.51	5.16	2.37
		1710	8.34	1.12	5.41	2.34
		4623	7.13	1.8	5.44	2.23
		5270	7.26	1.57	5.49	2.54

## Notes

1 Identity-first language has been used instead of person-first language in accordance with Kenny et al. (2016).

2 Over and above the contributions of AQ and concordance scores.
